# Towards the Rational Design of a Candidate Vaccine against Pregnancy Associated Malaria: Conserved Sequences of the DBL6ε Domain of VAR2CSA

**DOI:** 10.1371/journal.pone.0011276

**Published:** 2010-06-23

**Authors:** Cyril Badaut, Gwladys Bertin, Tatiana Rustico, Nadine Fievet, Achille Massougbodji, Alioune Gaye, Philippe Deloron

**Affiliations:** 1 Mother and Child Faced with Tropical Infections Research Unit, Institut de Recherche pour le Développement, UMR216, Paris, France; 2 Université Paris Descartes, Paris, France; 3 Département de Zoologie et Génétique, Faculté des Sciences et Techniques, Université d'Abomey-Calavi, Cotonou, Benin; 4 Centre de Santé Roi Baudoin de Guédiawaye, Dakar, Senegal; Walter and Eliza Hall Institute of Medical Research, Australia

## Abstract

**Background:**

Placental malaria is a disease linked to the sequestration of *Plasmodium falciparum* infected red blood cells (IRBC) in the placenta, leading to reduced materno-fetal exchanges and to local inflammation. One of the virulence factors of *P. falciparum* involved in cytoadherence to chondroitin sulfate A, its placental receptor, is the adhesive protein VAR2CSA. Its localisation on the surface of IRBC makes it accessible to the immune system. VAR2CSA contains six DBL domains. The DBL6ε domain is the most variable. High variability constitutes a means for the parasite to evade the host immune response. The DBL6ε domain could constitute a very attractive basis for a vaccine candidate but its reported variability necessitates, for antigenic characterisations, identifying and classifying commonalities across isolates.

**Methodology/Principal Findings:**

Local alignment analysis of the DBL6ε domain had revealed that it is not as variable as previously described. Variability is concentrated in seven regions present on the surface of the DBL6ε domain. The main goal of our work is to classify and group variable sequences that will simplify further research to determine dominant epitopes. Firstly, variable sequences were grouped following their average percent pairwise identity (APPI). Groups comprising many variable sequences sharing low variability were found. Secondly, ELISA experiments following the IgG recognition of a recombinant DBL6ε domain, and of peptides mimicking its seven variable blocks, allowed to determine an APPI cut-off and to isolate groups represented by a single consensus sequence.

**Conclusions/Significance:**

A new sequence approach is used to compare variable regions in sequences that have extensive segmental gene relationship. Using this approach, the VAR2CSA DBL6 domain is composed of 7 variable blocks with limited polymorphism. Each variable block is composed of a limited number of consensus types. Based on peptide based ELISA, variable blocks with 85% or greater sequence identity are expected to be recognized equally well by antibody and can be considered the same consensus type. Therefore, the analysis of the antibody response against the classified small number of sequences should be helpful to determine epitopes.

## Introduction

The severity of *Plasmodium falciparum* malaria is partially related to alterations of the IRBC induced by parasite proteins exported to the IRBC membrane during parasite development. Some of these proteins confer cytoadherence properties to IRBC, leading to parasite sequestration. The best characterized parasite adhesin is *P. falciparum* erythrocyte membrane protein 1 (PfEMP1) [1], encoded by the large polymorphic *var* gene family [2]. While mediating various binding activities and representing an important target for protective antibodies, PfEMP1 proteins undergo antigenic variation, contributing to parasite evasion of the host immune response.

Malaria during pregnancy, especially the first, is associated with strikingly adverse effects on foetal growth (reviewed in [3]). Pregnancy-associated malaria (PAM) parasites adhere to chondroitin sulphate A (CSA) [4], [5], a glycosaminoglycan expressed by the syncytiotrophoblast layer of the placenta. Several studies have shown that a single *var* gene, *var2csa*, is highly transcribed and expressed in both *in vitro* CSA-selected *P. falciparum* IRBC and parasites isolated from infected placentas [6], [7], [8], [9], [10]. During pregnancy, infected women may develop antibodies that inhibit IRBC binding CSA, and these women appear to be protected against PAM during subsequent pregnancies. Primigravidae lack these antibodies, which suggests that PAM parasites express novel surface molecules to which women have not been exposed previously [4], [11], [12], [13]. Antibody inhibition is not dependent on the geographical origin of parasites or sera [11], [14], [15], indicating that proteins mediating placental tropism are conserved or contain shared epitopes and that a vaccine against PAM is thus feasible. Furthermore, it has been shown that this immune response is at least partially directed against VAR2CSA and that antibody levels to VAR2CSA are sex- and parity-dependent. These antibodies are associated with reduced severity of PAM [16], [17], further supporting the role of VAR2CSA as the CSA adhesin in PAM.

Unlike other clinical malaria syndromes, PAM is associated with a single PfEMP1 variant expressed from *var2csa*. The *var2csa* gene is very particular within the *var* gene family: it is the only *var* gene that does not include CIDR domains, being composed of six DBL domains, three belonging to the ε type and three being unclassified (or X type) [18]. *Var2csa* is relatively conserved as the sequence of each DBL domain presents unusual homologies between parasite strains that are higher than other *var* genes [9], [19]. Nonetheless, the gene presents both highly conserved and highly variable regions, leading to a homology that varies from 54 to 94% between strains derived from different geographical origins [7], [20]. This variability is not be due to a shift in *var* expression, since only one *var2CSA* gene is present in most parasite strains, but rather to a mosaic structure [20], [21] generated by genetic recombination [22].

The DBL6ε domain has been described as the most polymorphic of the six DBL domains of *var2csa*, with an identity from 54 to 61% [23], [24]. As suggested by Bockhorst *et al.* [24], this may be partly related to a high level of epitope exposure, the presence of hidden epitopes arising from parasite selection and the high global variability exhibited by this domain. Epitopes present on the IRBC surface appear to be accessible to the solvent. All these criteria make these sequences of high interest for antibody targets.

During pregnancy, women acquire an immune response directed against the DBL6ε domain [17]. This domain is specifically recognized by plasma samples from pregnant women in a parity dependant manner, being higher with multigravidae than with primigravidae [25]. Antibodies raised against the DBL6ε domain from the FCR3 strain can bind the surface of erythrocytes infected by PAM isolates and also inhibit CSA binding [26]. A 30% sequence variation between two DBL6ε domains is sufficient to abolish the recognition by plasma samples from pregnant women [25], leading to immune evasion. Similar studies have also been performed on DBL3X [27], DBL2X [20], [23], [24], and DBL5ε [23]. The link between the variability of the DBL6ε domain and its immunogenicity [24], [25], [26] can be correlated with immune system evasion.

Although phylogenetic trees are useful to separate various *var* genes and to assign DBL and CIDR domain classes, global alignments of this type prove to be inappropriate for the study sequences within a given domain class [18]. Like Bockhorst *et al.* have shown for the DBL2X domain [24], we have developed an expanded alignment to study local alignments of variable blocks from DBL6ε domains. In particular, we developed a new graphical tool that permits discrimination between the most frequent sequences in the parasites. The objective of our analysis has been to classify the variable sequences in order to understand how variability is distributed over the DBL6ε domain. This classification could be helpful for the design of experiments that compare all sequences found for this domain.

## Results

### Amplification and sequences of DBL6ε from *var2csa*

Sequences of the DBL6ε domain from the *var2CSA* genes expressed by *P. falciparum* placental isolates were determined by transcription of mRNA purified from the parasitized blood cells. Oligonucleotides representing highly conserved sequences from the 3′ end of the DBL5ε domain (forward primer) and the 5′ end of the ATS domain (reverse primer) were designed to amplify the entire DBL6ε domain for sequence determination. From 24 placental parasite samples, 16 cDNA were amplified and cloned in pCR2.1 plasmid. For each isolate, a single sequence was obtained, except for the 2155 isolate from Thiadiaye, Senegal and the H852 isolate from Cotonou, Benin, which both gave two sequences. Lengths of the extended DBL6ε (with ID5, ID6, TM and a part of the ATS region) sequenced genes ranged from 1110 to 1414 nucleotides, depending of the isolate (Table 1).

**Table 1:** Summary of sequences from strains and isolates.

Isolates	Origin	Accession number	Length (bp)
3D7	Unknown	XM_001350379.1	1403
A4 (It4, FCR3)	Gambia	AY372123.1	1425
KMWII	Kenya	EF614230.1	1229
M24	Kenya	EF614231.1	1222
MC_var6	Thailand	AY372127.1	1387
MTS1	Burma	EF614227.1	ND*
P13	Mali	EF614232.1	ND*
PC49*	Peru	EF614233.1	ND*
T2C6	Burma	EF614225.1	1193
V1S	Vietnam	EF614224.1	1200
124-8	Sudan	EF614229.1	1197
HB3_1	Honduras	AANS01000262	ND*
HB3_2	Honduras	AANS01000295	1152
CYK57	Senegal	GQ848081	1371
CYK58	Senegal	GQ848082	1409
CYK24	Senegal	GQ848083	1374
CYK30	Senegal	GQ848084	1373
CYK32	Senegal	GQ848085	1387
CYK37	Senegal	GQ848086	1378
CYK48	Senegal	GQ848090	1378
CYK49	Senegal	GQ848087	1399
CYK 51	Senegal	GQ848088	1395
PAL09	Senegal	GQ848089	1414
PAL10	Senegal	GQ848091	1403
2155-3	Senegal	GQ848092	1386
2155-4	Senegal	GQ848093	1349
H839ABC	Benin	GQ848078	1159
H852B	Benin	GQ848079	1110
H852CD	Benin	GQ848080	1182

*Sequence too short; the carboxy-terminal region of the DBL6ε is missing.

29 strains and isolates DBL6ε accession number, their origin and length.

### Alignment, analysis, grouping, and classification of DBL6ε sequences

**Figure 1:**
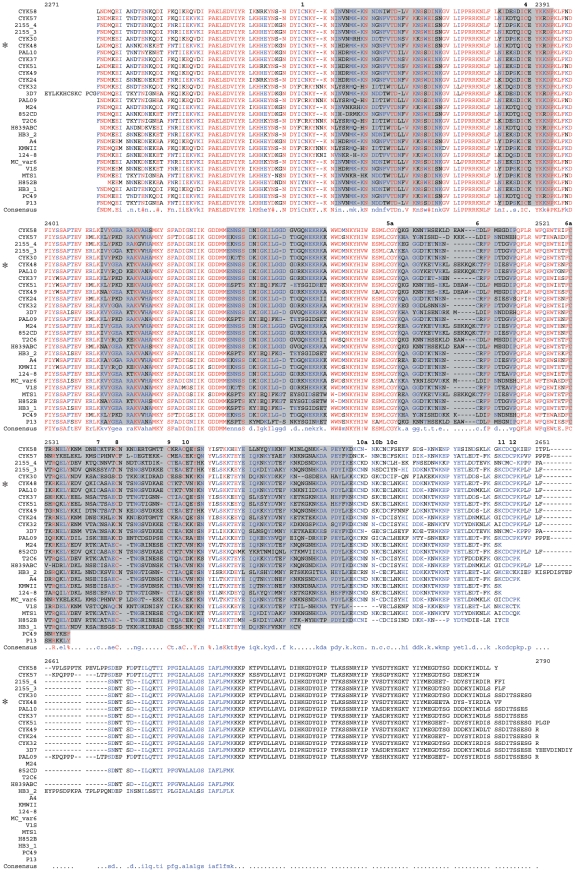
Alignment of the entire sequence of the DBL6ε domain from various isolates. With respect to the 3D7 PFL0030c residue numbering and combined the alignment, ID5 is located from M2287 to K2328, DBL6ε from G2329 to P2651, transmembrane domain from N2677 to P2710 and ATS from K2711 to the C-terminus. The highly conserved amino acids are coloured in red, the lowly conserved amino acids are in blue, and the highly variable amino acids are in black. The seven polymorphic blocks are highlighted in gray. At the top of the sequences, cysteine residues are numbered from 1 to 12 in bold. (*) indicates the sequence of the expressed and purified CYK48-DBL6ε domain. On the left, all strains and isolates are presented; all are listed in Table 1.

**Figure 2:**
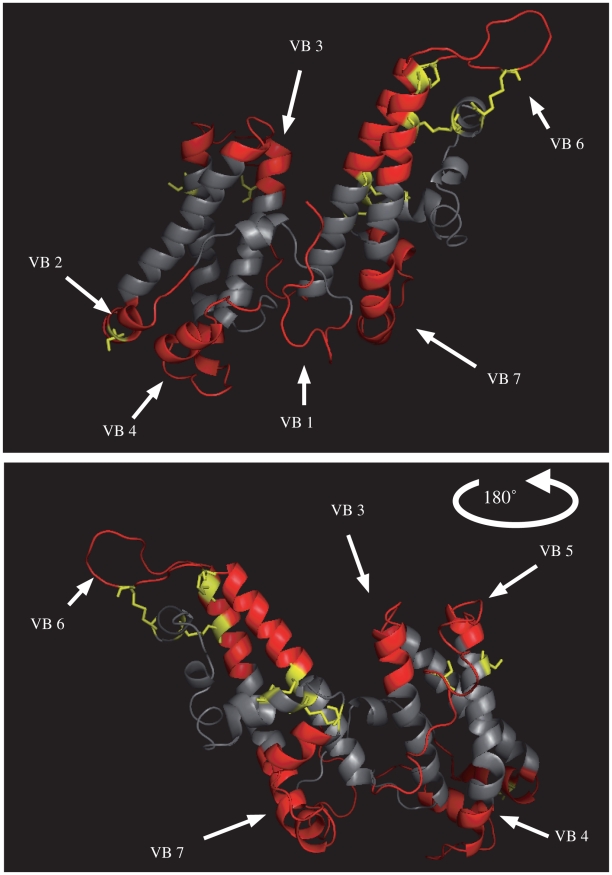
Structure model of CYK48-DBL6ε domain. This structure was performed with HHMM software using the DBL6ε (2WAU) domain of Kundrae *et al* as template. This model was used to determine the limits of variable blocks and to design relevant peptides. Cysteine residues are shown in yellow and variable blocks on the surface of the domain are in red. Conserved sequence is coloured in gray and is located in the core of the domain.

All DBL6ε domains were aligned using the 3D7 DBL6ε domain as a reference for residue numbering (Figure 1). Most sequences started at aa 2284. The DBL6ε domain extended from G2329 to P2651 and included 14 canonical DBL cysteine residues numbered in bold and shown in yellow in the structural model (Figures 1 and 2). The transmembrane segment extended from N2677 to P2710. The length of the DBL6ε domains varied from 269 to 303 residues for the isolates selected.

**Figure 3:**
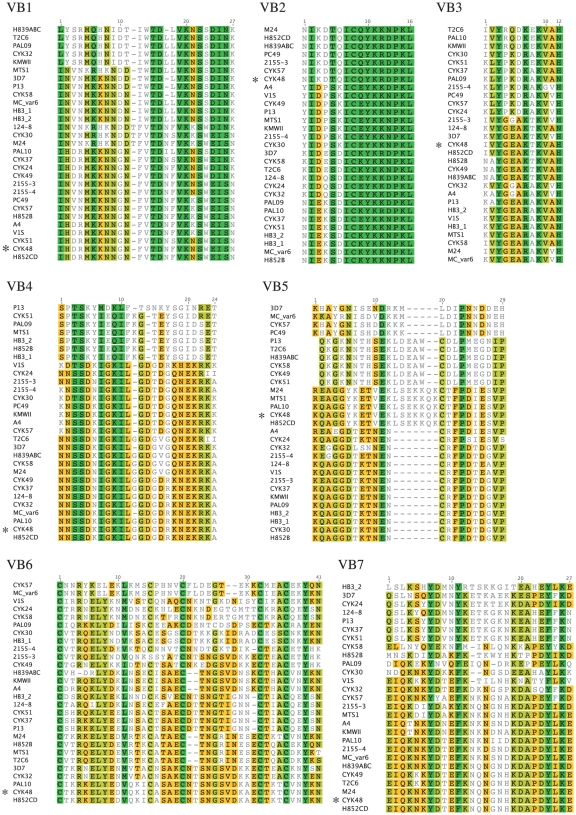
Alignment of the variable blocks. Alignments of variable blocks VB1, VB2, VB3, VB4, VB5, VB6 and VB7 shows that consensus sequence groups within each VB are limited in number. The sequence of CYK48-DBL6ε is indicated by (*). Green: 100% similar; khaki: 80 to 100% similar; Orange: 60 to 80% similar; Gray: less than 60% similar.

The global alignment (Figure 1) performed with all DBL6ε sequences (13 sequences from Senegal, 3 from Benin and 13 from data banks (Table 1)) showed a mean APPI of 67% and 97 identical amino acid residues (27%). The variability of the DBL6ε domain is not spread randomly along the entire sequence but is clustered in only a few blocks [24]. We clearly identified seven highly conserved blocks alternating with seven variable blocks, as also shown by others [24]. Three criteria were used to define the limits of these variable blocks (VB): (i) sequence must be variable, (ii) at least a few residues should be located in the loops of the CYK48-DBL6ε structural model and (iii) the corresponding VB are selected as solvent exposed in the structural model. If the variable amino acids were located in helices, as, for example, with the amino-terminal part (TKRKELYED) and for the carboxy-terminal part (TCVNYKN) of the VB6, they were not included as exposed to the solvent: these sequences were considered in the alignment, but not for the peptide synthesis. The seven VB were aligned independently of each other (Figures 3, VB1–VB7). Their limits are similar to those described by Bockhorst *et al* [24], except for VB2, VB6 and VB7. VB2 is much longer as it included five residues located in a structure that is exposed to the solvent in the model (Figure 2). VB6 was much shorter and in addition we identified a seventh VB from aa 2580 to aa 2606, corresponding to a loop of 14 amino-acid residues flanked by α-helices that is probably exposed to the solvent in the full-length VAR2CSA. As suggested by Bokhorst *et al.*, these polymorphic segments are concentrated in flexible loops [24]. Moreover, analysing VB in the structure model, no, or very little, secondary structure is observed.

A variable block (VB) is composed of the variable sequences found in one region on the surface of the DBL6ε domain from all isolates. For each VB, alignment and phylogenetic trees were generated. This revealed that each VB contained a small number of groups of similar sequence. Each group can be represented by a consensus sequence that is the average of all variable sequences present within this group. Thus each VB can be represented by a small number of consensus sequences.

**Figure 4:**
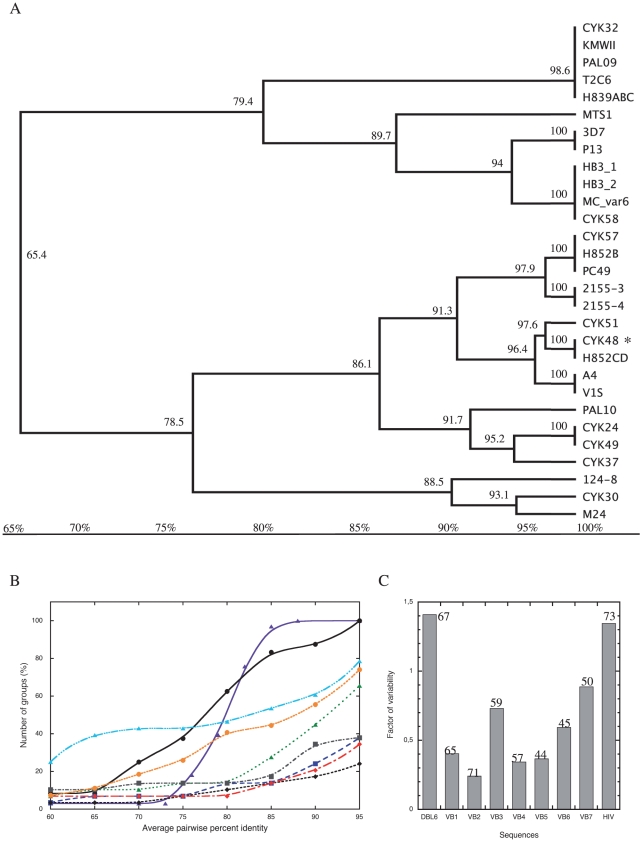
Graphical representation of the low VB variability. **A**: Trees were generated for each of the variable blocks in DBL6ε, branch annotations are the average percent pairwise identity (APPI) for VB1 sequences in this subtree. **B**: For various APPI (on the x-axis), branches (i.e. groups) are counted and marked on y-axis. Graphic representation of variability of the DBL6ε domain (black circle), the envelope protein of HIV (purple triangle), VB1 (blue square), VB2 (black diamond), VB3 (green triangle), VB4 (red diamond), VB5 (gray square), VB6 (light blue triangle) and VB7 (orange circle). In **C**, a histogram was constructed to determine the variability of the various blocks composed of more than one consensus sequence. HIV represents the histogram obtained with the Env glycoprotein of HIV. For comparison, global APPI of all the sequences are indicated at the top of each histogram.

An example is shown for VB1 (Figure 3 VB1, and Figure 4A), which is random coil in the structural model. Variability within these sequences is not due to random mutations within one consensus sequence but to two or more well-defined consensus sequences. Accordingly, the variability of the DBL6ε domain cannot be determined from its global APPI. A new graphic representation of the variability that improved the assessment of variability of these mosaic domains was developed as follows. A curve was plotted with the threshold for the APPI between sequences along the x-axis and the number of groups (i.e. number of branches counted, see Figure 4A) formed by these sequences at a given APPI threshold on the y-axis (Figure 4B). These curves show three components of the variability: (i) the number of consensus sequences within a VB; this number is found on the y-axis at the inflexion point (where the slope is maximum; see material and methods and Table 2), (ii) the variability of each consensus sequence within its own group; this variability increases with the difference between the ordinate at 95% and the ordinate at the inflexion point, and (iii) how well the groups are defined and are associated with representative consensus sequences; this is represented by the value of the abscissa at the inflexion point (Table 2): the level of similarity with the consensus sequence of the isolates classified in a group increases with this value. This representation was tested for the envelope glycoprotein of HIV (Env), which is a highly variable protein with random point mutations distributed along the entire gene [28]. This ‘reference curve of variability’ gave an inflexion point at 72% APPI, with a maximal amplitude as defined at Y (95%)–Y(60%). The set of DBL6ε sequences produced a curve typical of a highly variable sequence (Figure 4B). The inflexion point is observed at 65% APPI and its maximal amplitude is lower than that of Env. Only two groups are defined at 60% but 24 groups (from a total of 26 sequences) were obtained at 95% APPI.

**Table 2:** Characteristic values of curves.

Sequence	Y (60%)	Y (95%)	X (IP*)	Number of groups at 85% APPI
DBL6ε	2	24	65	20
Block 1	1	12	85	4
Block 2	2	7	85	4
Block 3	1	21	80	8
Block 4	1	10	80	6
Block 5	3	11	75	7
Block 6	7	20	90	14
Block 7	1	20	75	13

*Inflexion Point, where the slope of the curve is maximum.

Number of groups (Y value) at critical points (60 and 95%) and variability of consensus sequence (X value at the inflexion point (IP)). These values have been determined from the graphics on Figure 4B. The histogram was constructed using the following equation: [Y_norm_(95%)−Y_norm_(60%)]/X(IP) to describe the variability of a sequence where more than one consensus sequence is found.

The sequences of the seven blocks from the 29 isolates or strains were aligned. The VB1 sequence (2342–2368), with 25–27 residues, has no well-defined secondary structure in the structural model. The tree (Figure 4A) has two main branches corresponding to two widely divergent clusters of sequences. Moreover, at 85% APPI, four groups can be distinguished. The inflexion point (Figure 4B), is correlated with the sequence variability within each group. Like Bockhorst *et al.* [24], we identified VB2 (2382–2395) as a VB because it is present in the loop-αhelix-loop that should be accessible to IgG, and because of its high variability (Figure 3, VB2). However, we selected a longer sequence because an important mutation is present (N13 to D). In Figure 4B, only four consensus sequences occurred at APPI≥85%, and the variability of these sequences is very low (seven groups at 95%, Figure 4B). VB3 (2415–2427), with only 13 residues length (Figure 3, VB3) and structured as a small loop in the model, is much more variable; the inflexion point of this curve appears at low APPI (Figure 4B, 80%) and, moreover, the maximum amplitude is the largest of all the curves. This VB is the most variable and consensus sequences are not well defined since each consensus sequence presents many mutations (21 groups at ≥95%, Figure 4B). VB4 (2446–2470) is 25 residues in length and is an αhelix-loop-αhelix in our model (Figure 3, VB4 and 2). It shares an uncommon distribution of variability: six isolates from distant endemic areas can be grouped (VB1-4) in a separated cluster of identical sequences (CYK51 and PAL09 from Senegal, MTS1 from Burma, HB3_1, HB3_2 from Honduras and H852B from Benin). Only two groups are counted at the inflexion point (≥80%, Figure 4B) and 10 groups at 95%. Here again, the consensus sequences of this VB show low variability. VB5 (2488–2516), a random coil (Figure 2) formed by 22–29 residues, appears even more invariable. Among the sequences represented in Figure 3, VB5 shows a very special distribution: four very distant clusters of sequences with a very low variability can be easily distinguished within each group (group VB5-1 (86.1% PI; 76% Identical Sequence (IS)): KHAYGNISHDDKKXLDIPNNDNEH; group VB5-2 (98.7% PI; 96% IS): QKGKNNTHSEKLDEAWCDLPMEGDIP; group VB5-3 (95.9% PI; 93% IS): KQAGGYKETVEKLSEKKQKCTFPDIESVP, and group VB5-4 (85.1% PI; 59% IS): KQAGGDTKTNENCRFPDTDGVP). Each group has a strong signature with a very well defined consensus sequence, representative of almost all isolates found in endemic areas. Our graphical model cannot describe the low variability of these sequences from VB5 since the curve does not reflect an invariable loop (inflexion point at 75% APPI with seven groups). VB6 (2540–2563) has 38–41 residues (Figure 3, VB6) and forms an αhelix-loop-αhelix. We determined seven groups at ≥60% and 15 groups at the inflexion point (≥90%, Table 2 and Figure 4B). VB6 has a large number of consensus sequences but these present a very low variability. Even though 20 groups are counted at 95% APPI, the inflexion point is found at a high APPI percentage (90%). VB7 (2580–2606) of 25–27 residues (Figure 3, VB7) forms an αhelix-loop-αhelix and has the highest number of consensus sequences (inflexion point at 75% for 13 groups, Table 2 and Figure 4B) that are the most variable (20 groups at 95%). As with VB1, VB4 and VB5, four sequences of VB7-1 constitute a separate group (strains and isolates 3D7, CYK24, 124-8, CYK37, P13 and CYK51), with consensus sequences that are very different from the others within this VB.

Even though our analysis using a graphical representation of variability is more relevant than the percentage identity as is commonly used, it did not allow the extent of variability of VB5, VB6 or VB7 sequences to be determined. The number of groups defined at the inflexion point (Table 2) is not fully relevant with regard to the true variability. We therefore generated a Variability factor (VF) histogram (Figure 4C). The VF was calculated by dividing the difference between the number of groups defined at 60% and 95% APPI, and the inflexion point on the X axis (the threshold where variable sequences within one group formed share low variability, see Table 2). Ordinates were normalized by the number of the sequences analyzed (29 for VB1, VB2, VB3, VB4 and VB5; 28 for VB6; 27 for VB7; 26 for DBL6ε and 33 for Env). Because the VF is a combination of 3 independent variables characterising the curve in Figure 4B (see above), it is more relevant for analysis of the invariability of each VB. As positive control, we calculated the VF for the envelope protein ‘Env’ from HIV (1.35), which is a highly variable protein. The highest value of VF was calculated for the DBL6ε domain (1.41); the least variable sequence was VB2 (0.24). As we previously concluded, VB5 is not variable, having a low VF (0.37). The VF of VB3 (0.73) was very high, as was that of VB7 (0.89). With an VF of 0.59, VB6 is clearly not as variable as expected from its global APPI of 45%. Such apparently discrepant results arise from the fact that a large number of consensus sequences is indicated (Figure 4C), but these sequences are almost invariant. Here we have therefore developed two graphical approaches that together allow groups of sequences with more than one consensus sequence to be correctly analysed.

Finally, geographically distant isolates can share the same sequence, even with 100% APPI. Some sequences from VB1-4 are the same in Peru (PC49), Benin (H852B), and Senegal (CYK57)); the VB5-3 of strains from Kenya (M24), Burma (MTS1), Senegal (PAL10 and CYK48), and Benin (H852CD) have exactly the same sequence. Moreover, we did not find any country where all variable sequences share low variability and are represented by a single consensus sequence. These observations suggest that the groups of sequences and molecular signatures are independent of geographical origin and, consequently, that polymorphisms present in different parasite isolates evolved before the transcontinental spread of *P. falciparum*. These results should be confirmed with a larger number of sequences.

### Expression and purification of the CYK48-DBL6ε domain

**Figure 5:**
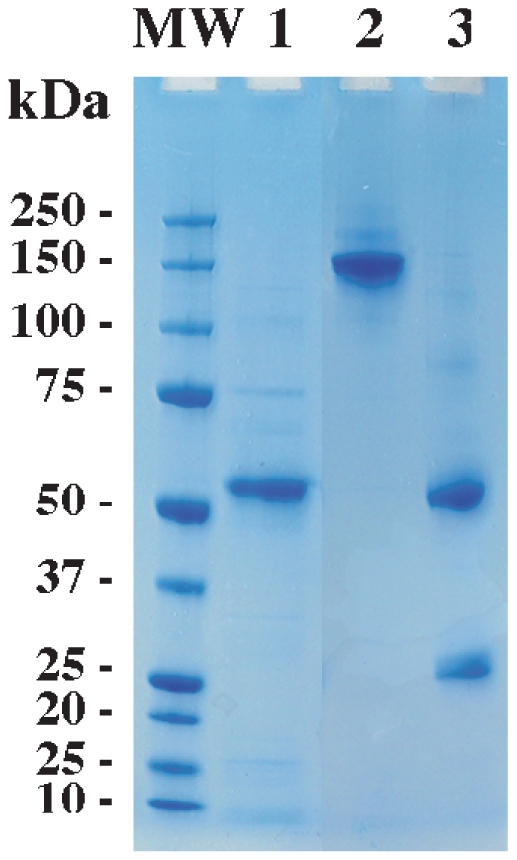
Purity of various proteins. CYK48-DBL6ε domain and antibodies observed after purification on 10% acrylamide gel (Invitrogen) stained with Coomassie blue. Reduced CYK48-DBL6ε domain (lane 1) after two purification steps and non-reduced or reduced antibody from protein G purification (respectively lanes 2 and 3). Molecular weight on left lane (Biorad).

Baculovirus was generated by homologous recombination between a plasmid carrying the DBL6ε gene and the linear baculovirus genome. After three virus amplifications and protein expression in insect cells, a two-step purification was performed. After passage over a cobalt gel column, a gel filtration purification step was carried out to remove remaining contaminants. The reduced 42 kDa domain (and non-reduced domain, data not shown) was observed on an acrylamide gel with an apparent molecular weight of 50 kDa and at least 90% purity (Figure 5 lane 1). A quantity of 400 µg of the domain was purified from 0.5 L of baculovirus-insect cells supernatant.

### Plasma samples recognition and CYK48-DBL6ε antigenicity

Experiments were designed to determine the percentage of plasma samples reacting against CYK48-DBL6ε and peptides based upon the seven variable blocks in this sequence. First, we investigated the reactivity of 80 plasma samples from primigravid or multigravid women. Plasma samples from non-exposed women and men were assessed as negative control. Plasma sample responses from exposed pregnant women were significantly higher than non-exposed men and women (Kruskal-Wallis test: p = 0.014). The response also increased with parity (p<10^−4^).

We purified and quantified IgG from 96 plasma samples. The concentrations of protein G-purified IgG varied from 0.23 to 0.98 mg/ml per 30 µl of plasma sample. Purity of the IgG is shown on Figure 5 where non-reduced IgG (150 kDa) is in lane 2, and reduced IgG (heavy chain, 50 kDa and light chain, 25 kDa) is in lane 3. We followed the reactivity against the DBL6ε-CYK48 domain with two positive and two negative plasma samples at various concentrations (data not shown). We determined the threshold concentration needed to produce a significant signal at 20% of the apparent avidity constant to be 0.0205 µg IgG/mL. This concentration was used in ELISA experiments with peptides mimicking the variable blocks.

### VB recognition and correlation between frequency recognition of sequences by plasma samples and prevalence of DBL6ε VB sequences within the population

**Figure 6:**
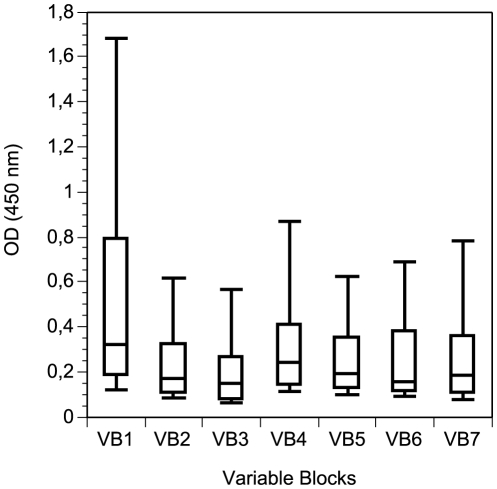
Reactivity of purified IgG against variable blocks. The level of purified IgG responses expressed as optical density (OD) units at 450nm against the various peptides is shown for women at delivery living in endemic area. The top, bottom, and middle lines of the box correspond to the 75th percentile, 25th percentile, and 50th percentile (median), respectively. The whiskers extend from the 10th percentile and top 90th percentile.

Our principal aim was to correlate the number of isolates sharing identical sequences of various VB at different APPI, and the percentage of plasma samples reacting against each VB. We performed ELISA experiments with synthetic biotinylated peptides. All were recognized by one or more plasma samples. No single peptide reacted with all purified IgG samples, and no single IgG sample reacted with all peptides, indicating that the responses are specific with very low experimental noise. Purified IgG from women and men from non-endemic areas recognized a mean (±SD) of 0.60 (±0.74) peptides, while primigravidae, secongravidae, and multigravidae recognized 1.79 (±1.17), 1.64 (±1.05), and 2.37 (±1.33) peptides, respectively, from the seven VB peptides tested by ELISA. As shown on Figure 6, the peptide from VB1 was recognised by more plasma samples (median = 0.32) than other (median = 0.15 to 0.25). VB1 and VB4 from CYK48-DBL6ε were the most frequently recognised of the seven VB studied from this isolate, with 63% and 35% of pregnant women plasma samples, respectively, recognising these sequences (Table 3). Sequences from VB1 and VB4 were present (at APPI>85%) in 48 and 46% of the parasite isolates, respectively (Table 3).

**Table 3:** Synthesised peptide sequences from CYK48-DBL6ε VB1 to 7.

VB	Sequences of peptides from isolate CYK48	% of isolates*	Reactivity of IgG§	Localisation
1	B-AhxGGIHDRMKKNNGNFVTDNFVKNSWEISN-Ct$	48	63	2342–2368
2	B-AhxGGNIKDTQICQYKRDP-Ct	29	20	2382–2395
3	B-AhxGGLKIVYGEAKTKVAHAM-Ct	14	13	2415–2427
4	B-AhxGGENNSSDKIGKILGGDGDRKNEKRKA-Ct	46	35	2446–2470
5	B-AhxGGKQAGGYKETVEKLSEKKQKCTFPDIESVP-Ct	17	16	2488–2516
6	B-AhxGGVQKICASAECNTSNGSVDKAECTK-Ct	11	21	2540–2563
7	B-AhxEIQKNKYDTEFKNQNGNHKDAPDYLKE-Ct	40	29	2580–2606

*Identical at 85% of average percentage pairwise identity.

§ In % on 80 purified IgG.

$ B: Biotin; Ct: Carboxyterminal.

Sequence of variable blocks from CYK48-DBL6ε. For example, the number of isolates identical to CYK48 VB1 determined from Figure 4B. At the branch where APPI>85% (here 86.1%), 14 strains are counted (for a total of 29 strains: 48% of them have the same sequence at APPI>85%). The other VB values were determined as the same way.

**Figure 7:**
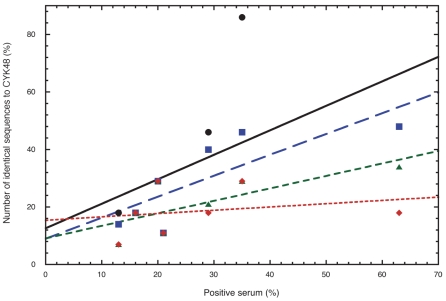
Graphic representation of the variability tolerated for a sequence that remains dominant epitope at various identity percentages. Experimental responses of purified IgG against the VB are shown in percent on the x-axis and are summarised in Table 3 (Reactivity of IgG). The occurrence of each VB sequences in the population was counted. For example, in Figure 4B at APPI>85% (here 86.1%) we counted 14 strains (or 14/29*100%). In Figure 4, the number of sequences identical to CYK48 (indicated with*) were counted. Percentage of isolates with the same sequence as CYK48 for each VB is indicated on y-axis at 80% (black circle), 85% (blue square), 90% (green triangle), or 95% (red diamond) APPI. For example, see values for APPI = 85% in Table 3 (% of isolates). Experimental distribution around the linear curve is linked to the APPI tolerated to have an identical IgG response to two sequences. The best Pearson's r (0.74) for a linear model is calculated for 85% APPI. This graph indicates a cut-off of 85%. In our ELISA, two sequences sharing this APPI will have the same antibody response.

We searched for a correlation between the frequency of VB sequence recognition by the plasma samples and the prevalence of DBL6ε VB sequences within the parasite population. VB1 to VB7 of CKY48-DBL6ε were recognised by 63, 20, 13, 35, 16, 21, and 29% of plasma samples, respectively (Table 3). The percentage of isolates sharing each VB sequence with CYK48-DBL6ε at various APPI (80, 85, 90, and 95%) was plotted in Figure 7. For VB1, at thresholds of 80, 85, 90, 95% of APPI, respectively, 54, 54, 39 and 18% of isolates shared the same sequence. We determined these values for the remaining VB and plotted the threshold for the average identity between sequences for each loop (x axis) against the percentage of plasma samples that recognise the CYK48-DBL6ε domain (y axis) and the curve was fitted by linear regression. The correlation coefficient (Pearson's r) was determined for all VB at four APPI thresholds (80, 85, 90, and 95%). The highest correlation coefficient (r = 0.74) was obtained at 85% PI, suggesting that, in our assay, two women infected by two parasites sharing a VB with at least 85% APPI should have plasma samples that react to the consensus sequence tested. This observation allows a classification of the VB sequences and to group those that share at least 85% of APPI. This classification allows the consensus sequences representing groups of sequences to be identified. In our ELISA assay, the reactivity of plasma samples to a given consensus sequence of a group was representative of responses to each of all sequences from this group.

Once this threshold was determined, we re-examined the trees and groups formed, counted the number of groups and determined the consensus sequences that were representative of all VB sequences found in endemic areas. This classification allowed us to determine that, for VB1, 2, 3, 4 and 5, only four or five consensus sequences with at least 85% APPI are representative of each VB found in the DBL6ε isolates sequenced (Table 4). For example, four consensus sequences were identified for VB1 that represent groups formed with 98.6, 89.7, 88.5, and 86.1% APPI. These groups represent 17, 24, 10 and 48% of the parasites studied, respectively, and thus essentially 100% of the parasite sample. The other VB showed similar characteristics, suggesting that these sequences effectively represent all existing sequences. Groups formed by VB6 and VB7 are less relevant as eight and three consensus sequences are representative of 80 and 63% of the parasite isolates, respectively.

**Table 4:** Consensus sequences found in all VB of the DBL6ε domain.

VB	Sequences	%APPI§	%*	%&
VB1-1	LYSRMQHNIDTIWTDLLVKNSSDINK	98.6	17	100
VB1-2	INVNMKKNNDNIWTDLLVKNSSDINK	89.7	24	
VB1-3	INVNKRHKNDDTFVTDNFVKKSWEISN	88.5	10	
VB1-4	IHDRMKKNNGNFVTDNFVKNSWEISN	86.1	48	
VB2-1	NIKDTQICQYKRDPKL	96.4	24	100
VB2-2	YIDPSKICEYKKNPKL	95.3	28	
VB2-3	KIDZSDICEYKKDPKL	87.1	21	
VB2-4	NIEKSDICKYKKNPKL	93.1	28	
VB3-1	IVYRQDKEKVAN	94.4	10	93
VB3-2	KLYPKDRAKVVH	84.1	24	
VB3-3	NVYGEAKTKVAH	84.1	24	
VB3-4	KAYGGARAKVVH	91.7	7	
VB3-5	KVYGEARAKVAN	86	28	
VB4-1	SPTSKYIEQIFKGTEYSGIDSET	85.7	24	100
VB4-2	KNSSDKIGKILGDTDGQNEKRKK	86	31	
VB4-3	ENNSSDNIGKILGGDGDRKNEKRKA	88.3	45	
VB5-1	KHAYGNISHDDKKKLDIPNNDNEH	86.1	10	97
VB5-2	QKGKNNTHSEKLDEAWCDLPMEGDIP	98.7	21	
VB5-3	KQAGGYKETVEKLSEKKQKCTFPDIESVP	95.9	17	
VB5-4	KQAGGDTKTNENCRFPDTDGVP	85.1	48	
VB6-1	CTKRKELYEDVQKICASAECNTSNGSVDKAECTKTCVNYKN	98.4	11	80
VB6-2	CTRRNELYENMVTACNSAKCNTSNGSVDKKECTEACKEYSN	92.7	7	
VB6-3	CVTRQELYDEVRTKCATAECTNGRINESECTQACDKYKN	84.2	15	
VB6-4	CSHRKKLYEELNSACTSAECDTTNGTIGNNCTIACQEYSN	88.3	15	
VB6-5	CVTRQELYDKLNSECISAECTNGSVDNSKCTHACVNYKN	89.7	11	
VB6-6	CVTRQELYNDVKSACESGSCDTKKGKIDRADCESSCNKYKN	100	7	
VB6-7	CTRRNELYKNMDNECKSAKCNKNDEGTGMTTCKRACQEYSN	90.2	7	
VB6-8	CNNRYKELEKLKMSCPHNVCFLDEGTEKKCIEACEKYQN	94.9	7	
VB7-1	QSLKSYYDVNYKETKGKNKEAHEFFKN	94.4	15	63
VB7-2	EIQKNKYDTEFKNQNGNDKDAPDYLKE	86.3	41	
VB7-3	EIQKDIYDAKFKNKNGNHKDAPDYIKE	85.2	7	

§ average pairwise identity for sequences within this consensus group. Sequences with 85% or greater pairwise identity were considered the same consensus group based on ELISA reactivity in Figure 7.

*Percentage of parasites presenting a consensus sequence with average percent pairwise identity.

& Percentage of parasites presenting one of the listed consensus sequences at this given VB.

Finally, we tried to correlate the response of plasma samples with the VB sequences of the DBL6ε domain isolated from the same woman (i.e. matched plasma sample and sequence). The reactivity of 11 plasma samples against the VB5 from CYK48-DBL6ε was assessed (see Table 5 for loop 5). The matched plasma sample (woman CYK48) did not react against the DBL6ε-CYK48 domain. Plasma sample from patient PAL10, whose parasite had exactly the same VB5 sequence, reacted strongly with CYK48 VB5 sequence. This response variability was observed in many individuals, and did not appear to be related to parity.

**Table 5:** Sequences and percentage of identity of loop 5 from isolates.

Isolates	Sequences of VB number 5	%APPI*	IgG reactivity$	Gestity
CYK48	KQAGGYKETVEKLSEKKQKCTFPDIESVP	100	−	2
PAL10	KQAGGYKETVEKLSEKKQKCTFPDIESVP		++	3
PAL09	KQAGGDTKTNENCRFPDTDGVP	80.5	−	6
CYK32	KEGGGDLSNNENCRFPDTDGVP		−	1
CYK30	KQAGGDTKTNENCRFPDTDGVP		++	1
CYK24	KQAGGDTKTNENCRFPSIESVS		−	1
CYK37	KQAGGDTETNENCRFPDTDGVP		+	4
CYK58	QKGKNNTHSEKLDEAWCDLPMEGDIP	100	+++	2
CYK49	QKGKNNTHSEKLDEAWCDLPMEGDIP		++	2
CYK51	QKGKNNTHSEKLDEAWCDLPMEGDIP		−	7
CYK57	KHAYGNISHDDKKKLDIPNNDNEH	86.1	++	1

*Average percent pairwise identity.

$ To the VB5 of CYK48-DBL6ε.

Sequences of the various VB5 of isolates to be compared with their IgG reactivity.

We initially hypothesised that two individuals infected by parasites sharing at least one identical VB sequence should present IgG that recognize the DBL6ε domain. In fact, recognition of a given VB by a plasma sample was not related to the DBL6ε sequence encountered at delivery. Two hypotheses may account for these observations. First, an early infection in pregnancy may induce an effective antibody response that, in turn, leads to parasite clearance. Second, an infection occurring very late in pregnancy will not be able to induce a specific antibody response at delivery. Both possibilities would lead to a lack of correlation between the DBL6ε sequence present at delivery and the specific antibody response. Such findings are in agreement with previous work [29], [30], which showed that women exposed to malaria do not acquire VSA-PAM-specific antibodies earlier than halfway through their first pregnancy. Similar phenomenon can be transposed to multiparous women, in which multiplication of parasites requires that these parasites express new epitopes unknown for this woman.

## Discussion

Many studies have addressed the variability of VAR2CSA sequences. Although most have studied variability at the level of the entire domain, a few studies have investigated the basis of variability of DBL domains in depth [20], [23], [24], [27]. Here, we have described DBL6ε, the most variable of the six DBL domains of the VAR2CSA adhesin. This domain is of particular interest and relevance as it is subjected to an uncommonly high selection pressure. After alignment and tree analysis of complete DBL6ε domain sequences, no consensus sequence is evident. As already observed, further detailed DBL6ε sequence analysis reveals that these domains are mosaics constituted of highly conserved blocks alternating with seven highly variable blocks on the surface of the structure model. Local alignment analysis of these variable blocks revealed that their variability is not due to random mutations on a single consensus sequence. In each VB, four or five groups are represented by a highly conserved consensus sequence that emerges from alignments, their permutation generating the high DBL6ε variability. Within these groups, variability is introduced by insertions, deletions or by conservative mutations.

We performed alignment and tree building to elaborate a new approach to analyse the variability of sequences presenting more than one consensus sequence. Graphical and histogram representations, combined with ELISA experiments, allowed identification of these groups and their representative consensus sequences, and evaluation of their variability. Most VB are weakly variable since only a few mutations were observed within their consensus sequences, as observed for VB5. For VB6, eight consensus sequences that represent only 80% of the parasites were counted but only few residues are mutated within each group. VB3 is the most variable since five consensus sequences were found with low APPI (Table 4). The high variability apparent in the DBL6ε domain is due to three factors: the mosaicity of its sequence, the presence of various consensus sequences and the low variability within the groups. We determined the most frequent consensus sequence in the population. The study of all consensus sequences of each VB will allow determining which one should be dominantly recognised by antibody. Finally these results should simplify future research of dominant epitopes by reducing the number of possible sequences to be studied.

We studied the reactivity of plasma and purified IgG from pregnant women against a recombinant DBL6ε domain from a placental isolate and seven peptides mimicking the variable blocks from this domain. Most plasma samples from infected pregnant women reacted with the recombinant domain. All peptides were recognised by at least one of the plasma samples and most plasma samples recognised at least one peptide. Thus, synthetic peptides were able to mimic at least linear epitopes present within VB. Although the presence of conformational epitopes in the DBL6ε domain cannot be excluded, this does not invalidate our data obtained with peptides. This study demonstrates that VB are immunogenic and recognized by maternal antibodies. The likely cause of variability of the VB comes from a selection pressure due to their accessibility to antibodies, as suggested by the DBL6 model.

We analysed the occurrence of VB consensus sequences from the DBL6ε domain, hypothesising that this distribution does not vary geographically. We compared the prevalence of each sequence in the parasite population with the percentage of pregnant women plasma samples, from Benin and Senegal, recognizing it. We found a strong correlation tolerating at least 85% of APPI in our ELISA assay. At this threshold, these sequences could be described by a single consensus sequence representing the group and can thus be considered as an identical epitope. A high number of plasma samples and sequences originating from a given endemic region should increase the accuracy of the data. Additional data relating sequences and their recognition by plasma samples should confirm whether antigenic sequences vary geographically.

These sequences within the domain present neither secondary structure nor well defined highly compact structure, as suggested by Bockhorst *et al.* [24]. Some VB structures could not be traced in the DBL6ε-3D7 crystal structure: any two VB lacking an energetically stable structure cannot significantly effect on each other. Moreover, all VB are very distant from each other in the DBL6ε structure, preventing interactions between them. No structural changes can be transmitted from one VB to the other because most cysteine residues forming cystine bridges lock the structure, and prevent structural deformations. The presence of two consensus sequences in two VB are never correlated (perhaps due random permutation of VB). All these indications affirm that the VB are completely independent within the DBL6ε domain.

Because each VB presents a maximum of four to five independent consensus sequences (Table 4), the seven VB sequences of each sequenced DBL6ε domain can be represented by the VB sequences from only five DBL6ε domains. Consequently, studying the immunogenicity of five domains should represent all DBL6ε tolerating at least 85% APPI.

Many studies by different groups have given divergent results. This apparent discrepancy may result from the fact that the FCR3 and 3D7 strains used by most laboratories differ from wild isolates [25] as well as they differ from each other; the resulting immune response may therefore diverge. We confirmed that two DBL6ε from two strains or isolates are highly unlikely to share the same sequence. Since this variability leads to immune system evasion, it is difficult to compare studies performed with DBL6ε domains from different isolates because our results suggest that any given DBL6ε induces antibodies that react only with sequences from each of the seven VB of this isolate. As each VB exhibits only four or five distinct consensus sequences, the likelihood that a VB expressed by a new parasite has been previously encountered by the individual increases with the number of preceding infections. Each new infection will move the VB antigenic repertoire towards completion, but given the reduced number of possible consensus sequences at each VB level, the entire repertoire should be rapidly encountered after first pregnancies, in line with the frequent observation of anti-VAR2CSA antibodies in multiparae.

Our approach is one of the first to study short sequences from a DBL domain. The first studies aimed to classify and group DBLα involved in rosetting phenotypes [21], [31] and DBL2X from *var2csa* [24]. We did not compare all DBLε domains within parasites since DBL6ε was amplified with a downstream primer located in the ATS of the transcribed *var2csa* gene in parasites isolated from infected placentas. Like Bull *et al.* [31] with PoLV 1–4 sequences, we used positions of ‘limited variability’, described as highly variable sequences, to classify DBL6ε sequences. This classification should help for future experiments on a restricted number of potential immunogenic sequences.

A relationship between the occurrence of given consensus sequences in the parasite population and the frequency of reacting plasma samples in the human population from the same area indicates that these consensus sequences are still expanding. At the end of the expansion of a sequence, most people have acquired antibodies against strains sharing a given epitope, leading eventually to the disappearance of the strains expressing this sequence in pregnant women. Therefore, an imbalance between the proportions of positive serum and the occurrences of sequences should be observed.

These findings should allow, in future experiments, testing the potential dominance of epitopes formed by a small number of consensus sequences found in all variable blocks with PAM-specific IgG in order to ascertain the sequences representing dominant epitopes. A combination of antibody may be required to be protective, eventually involving antibodies against DBL6ε but also against other DBL of VAR2CSA.Finally, this should aid the identification of the most antigenic and immunogenic epitopes on the surface of the DBL6ε domain that would constitute an effective pregnancy-associated malaria vaccine. The approach described here can be applied to the other DBL domains of VAR2CSA to determine if these conclusions also extend to other regions of this variant.

## Materials and Methods

### Sample collection

Samples were collected in Senegal and Benin as part of studies of *P. falciparum*-infected pregnant women in Thiadiaye in 2001 [32], and Pikine-Guédiawaye in 2003–04 [33], Senegal, and in Cotonou, Benin in 2006–2007 [34]. At parturition, malaria infection was assessed by examination of placental blood smear by microscopy. Total RNA from IRBC containing trophozoite/schizont stage parasites from placental blood was prepared using organic extraction from Trizol [6]. Plasma samples were isolated and stored at −20°C until use. The National Ethics Committee of the Senegalese Health Ministry and the Science and Health Faculty Ethics Committee in Benin approved these studies. Written informed consent for data and blood collection and storage was obtained from all subjects before enrolment in the study. Thick and thin smears of blood from both the maternal side of the placenta and peripheral venous blood were Giemsa stained and examined for the presence of parasites, pigment.

### Cell lines

Insect cells (*Spodoptera frugiperda* 9 [Sf9]) were maintained at 27°C in TC-100 medium (Invitrogen) containing 2 g/mL glucose, yeastolate (Invitrogen), and 5% fetal calf serum (FCS; Eurobio). High Five (HF) cells (Invitrogen) were grown at 27°C in Insect XPress medium (Cambrex).

### DBL6ε domains sequenced from placental isolates

The various DBL6ε domains were amplified from cDNA of placental isolates after RNA extraction and reverse transcription, as previously described [6]. Primers were designed to amplify all DBL6ε domains from *var2csa*. The forward primer containing the *BamHI* sequence was complementary to a highly conserved DNA sequence within the 3′ part of the DBL5ε domain (DBL6F: 5′ CATTGTTCTAAATGTCCGTGTGGA 3′). The reverse primer containing the *PstI* sequence was complementary to a highly conserved DNA sequence within the 5′ region of the ATS domain (5′ DBLRev3: TTCGGAGGAAGTTATATCAGAGGA 3′). The high fidelity Taq polymerase phusion (Finnzymes) was used with the following PCR program: 94°C–7min, (94°C–30sec, 49°C–30sec, 68°C–2min) 5 times, and (94°C–30sec, 57°C–30sec, 68°C–2min) 30 times, then 68°C–7min. After digestion and insertion in the pCR2.1 plasmid, the insert of four clones was sequenced using a sequencing kit big dye terminator v1.1 (Applied biosystems). The chromatograms were obtained with an ABIprism 3100 sequencer. The software multalin (http://bioinfo.genotoul.fr/multalin/multalin.html) was used with the default settings to align the DNA sequences of the DBL6ε domains.

### DBL6ε sequences analysis: protein alignment, phylogenetic tree, and pairwise identity

Sequence alignment of was performed with ‘genious 4.6.2’ software, the plugin ‘muscle’ (http://www.geneious.com/) and BLOSUM62 matrix. Since the entire sequence of the domain was required for a good alignment, only 26 sequences for which the complete sequence was available were used. For most of the blocks, 29 sequences were available. Trees were generated with the same software using the genetic distance model of Jukes-Vantor with the ‘Unweighted Pair Group Method with Arithmetic mean’ (UPGMA) tree build method. Average percent pairwise identity (APPI) was determined for each group. At various APPI thresholds (≥60, 65, 70, 75, 80, 85, 90 and 95%), values were plotted on x-axis against the number of groups counted at each of these values on the y-axis. The number of sequences aligned was variable: for the overall DBL6ε domain sequence, 24 sequences were used, while sequences from blocks 1–5 came from 27 isolates, and those from block 6–7 came from only 26 isolates. All accession numbers are given in Table 1. Transmembrane domains were detected with the software http://www.cbs.dtu.dk/services/TMHMM-2.0/.

From the tree in Figure 4A, a curve was constructed by counting the number of sequence groups that formed when the sequences were clustered into groups by their APPI. As an example, for VB1 (blue squares) less than 60%, all sequences are grouped because the global APPI identity is 65.4%. There is a total of 29 sequences in the tree for VB1, so the maximum number of groups is 29 (all sequences form their own group). The y-axis gives the percentage of possible groups for a given APPI threshold, which at the 60% pairwise cut-off is 1/29 (or 3.5%). At the other end of the plot for 95% identity there are 11 groups for VB1 (11/29 or 38%). The number of groups can be derived by sectioning the tree vertically, where all branches cut are either terminal branches or have APPI>95%. The curve is generated by computing the group percentage for a variety of thresholds on APPI. This method is used to draw the curves for all VBs, the whole DBL6ε domain, and the HIV env.

Figure 4B is a synthesis of the trees constructed for all VB. These curves have been drawn to identify true sequence variability that are derived from more than one identical ancestral sequence. APPI are plotted on the x-axis and the number of groups counted at each APPI value is plotted on the y-axis. At APPI<the inflexion point, branches are very distant and the variable sequences are very different. At APPI>the inflexion point, sequences share a low variability between themselves and can thus be represented by a single average sequence.

Figure 7 was constructed as follows: for VB1, for example, the number of sequences identical to CYK48 was counted. On the branch where APPI>85% (here 86.1), 14 strains (for a total of 29, i.e. 48%) are in the same group, represented by a single consensus sequence. The calculated values for all VB are on the y-axis (see example for VB1 in Table 3, % of isolate at APPI>85%). The percentage of positive IgG against the VB are on the x-axis. We compared the curve fit (linear regression) for the four curves following Pearson's r. We BLASTed these VB sequences and found no human protein with a high score and low E value.

### Construction, expression, and purification of the recombinant CYK48-DBL6ε domain and peptides design and synthesis

The CYK48-DBL6ε domain sequence was cloned from a Senegalese placental isolate and DBL6ε was obtained by PCR with the following specific primers: FID5AN: 5′ CAGTCGGATCCTATGCAAGAAATAGCTAACAATAAAG 3′, and RID6KS: 5′ GTCGCCGCTGCAGTTAATGGTGATGGTGATGGTGGTCCTCCAGAGTTTCATAAGGATTT 3′ on the plasmid pCR2.1 carrying the ID5-DBL6-ATS DNA fragment sequenced. After *BamHI* and *PstI* digestion, the DBL6ε DNA PCR product was ligated into the pAcGP67A plasmid (BD bioscience) and fused with the GP67 secretion signal sequence at the amino-terminus and with a C-terminal hexa-histidine tag. As described in the manufacturer's protocol, the construct was co-transfected with the baculovirus linear DNA (BD bioscience) into Sf9 cells in the presence of Cellfectine (Invitrogen). Amplification of viruses was done in Sf9 cells and HF cells were used for protein expression. The recombinant protein was secreted in the XPress medium, harvested, and dialyzed against 20 mM Tris (pH 7.5) and 500 mM NaCl. After equilibration with dialysed buffer, the Talon gel (Clontech; BectonDickinson) was incubated with the dialysed protein, packed in a HR5-5 GE Healthcare column, washed with 20 ml of dialysis buffer and eluted with the same buffer plus 0.3 M imidazole. Fractions were pooled and concentrated in an YM-10 Centricon column (Amicon; Millipore) and a volume of 2mL was injected at 0.6 ml min^−1^ on a high-load 16/60 Superdex 200 column (GE Healthcare) pre-equilibrated in 20mM Tris–HCl (pH 7.5), 0.5M NaCl, and the absorption was followed at 280 nm. Fractions from 75 to 82 ml were collected, pooled and concentrated. Concentration was estimated using the calculated extinction coefficient (1.553 mg/mL/OD_280_) determined with http://www.expasy.ch/tools/protparam.html web site. The calculated mass of the CYK48-DBL6ε domain is 42.072 kDa and the theoretical pI is 8.28. Five µg of the purified domain were loaded onto a 10% acrylamide gel run at 200V stained with Coomassie blue, or transferred onto a nitrocellulose membrane and incubated with an anti His-tag HRP coupled antibody (Roche). The revelation was done using of an ECL kit (GE Healthcare).

Biotinylated peptides allowed coating of a constant quantity and steric hindrance was minimised by the presence of an arm of an ε-aminohexanoic acids (Ahx) and two glycin residues (except for peptide representing VB7 without glycin), permitting a better epitope presentation and a potentially more efficient antibody recognition. All peptides were synthesised by Genepep.

### Model of DBL6ε structure

The 3D-model was calculated with HHpred (http://toolkit.tuebingen.mpg.de/hhpred) and the sequence of CYK48-DBL6ε domain was aligned within the structure of DBL6ε (2WAU) [35] with default settings. Pictures have been generated with MacPyMOL.

### ELISA responses of plasma samples or purified IgG against CYK48-DBL6ε or peptides

The specific response directed against the purified recombinant CYK48-DBL6ε domain was assessed in plasma samples from 52 multigravid and 28 primigravid women from Senegal, eight plasma samples from men, and eight from women, both living in non-endemic area. 96-well plates (maxisorp, NUNC) were coated with 100 µL of 0.5 µg/mL of antigen diluted in PBS and incubated over night at 4°C. After 3 washing steps with washing buffer (1% Tween 20 in PBS), wells were blocked with blocking buffer (2% BSA, 0.1% Tween 20 in PBS) and after 3 washing steps, they were incubated with 100 µL of human plasma samples (1∶400) diluted in blocking buffer, followed by horseradish peroxidase-conjugated anti–human IgG (1∶4000). TMB was added and the reaction was stopped after 15 minutes with 50µl of 0.25M H_2_SO_4_. The optical density (OD) was read at 450 nm, and final values were obtained by subtracting the average OD of duplicate wells from that of the corresponding blank wells coated with BSA 0.5 µg/mL in PBS.

We performed a similar ELISA assay with various IgG concentrations to check the response of positive and negative plasma. The results were analyzed using the Kaleidagraph software according a hyperbolic saturation curve to determine the apparent avidity constant (K_app_) using the following equation: (OD_max_−OD_min_)*(C/K_app_+C)+OD_min_ where OD was determined at maximum and minimum purified IgG concentrations and C was the IgG concentration used. ELISA assays were carried out in duplicate. Ninety-six purified IgG responses against 7 peptides mimicking the seven variable blocks present within the CYK48-DBL6ε domain were measured. IgG was purified by incubating 30 µl of plasma samples on protein G columns to prevent from saturation and to determine the exact amount of IgG present in the plasma. Ninety-six-well plates coated with streptavidine (NUNC) were incubated with 200µL of blocking buffer (0.5% milk, 0.1% Tween 20 in PBS) at room temperature for one hour and washed 3 times (PBS, 0.1% Tween 20). A volume of 100 µL containing 3.10^−11^ moles of each peptide diluted in PBS was incubated for one hour at room temperature followed by 3 washing steps. One hundred µL of IgG at a concentration of 0.0205 g/L and diluted in blocking buffer were incubated for 90 min at 18°C. After 3 washing steps, horseradish peroxidase-conjugated anti–human IgG (1∶3500) was incubated for one hour at room temperature. TMB was added and reaction was stopped by adding with 30µl of 0.25M H_2_SO_4_.
